# Assessment of biochemical and hematological profiles in outpatients with serological evidence of SARS-CoV-2 infection

**DOI:** 10.3389/fmed.2026.1778397

**Published:** 2026-03-09

**Authors:** Samuel Salazar-García, Eunice Lares-Villaseñor, Juan Manuel Vargas-Morales

**Affiliations:** Laboratorio de Análisis Clínicos, Facultad de Ciencias Químicas, Universidad Autónoma de San Luis Potosí, San Luis Potosí, Mexico

**Keywords:** dyslipidemia, HDL cholesterol, liver enzymes, SARS-CoV-2 seroprevalence, unvaccinated population

## Abstract

**Background:**

SARS-CoV-2 infection is known to induce systemic biochemical and hematological alterations, particularly in hospitalized patients. However, data regarding subclinical changes in ambulatory, non-hospitalized individuals—especially in Latin American populations—remain limited.

**Objective:**

To compare biochemical and hematological parameters between ambulatory adults with and without serological evidence of prior SARS-CoV-2 infection, in order to identify potential subclinical alterations associated with previous virus exposure.

**Methods:**

A cross-sectional study was conducted in 201 ambulatory adults from central-northern Mexico between August and December 2020. Anti–SARS-CoV-2 IgG and IgM antibodies were detected using two independent lateral flow immunochromatographic assays. Participants were classified as seropositive only when both assays showed concordant reactivity. Biochemical and hematological parameters were compared between seropositive and seronegative individuals using appropriate parametric or non-parametric tests.

**Results:**

Overall seroprevalence of anti–SARS-CoV-2 antibodies was 22.3%. No significant differences were observed in hematological parameters between seropositive and seronegative participants. In contrast, seropositive individuals exhibited significantly lower high-density lipoprotein cholesterol (HDL-C) levels compared to seronegatives (median: 40.5 vs. 45.3 mg/dL; *p* = 0.009), with significance maintained in females. Liver enzymes were significantly higher among seropositive subjects, including aspartate aminotransferase (AST) (median: 30 vs. 24 U/L; *p* = 0.007) and alanine aminotransferase (ALT) (median: 32.5 vs. 20 U/L; *p* = 0.001). These differences were more pronounced in males. No significant differences were found in glucose, total cholesterol (TC), low-density lipoprotein cholesterol (LDL-C), triglycerides (TG), alkaline phosphatase (ALP), or total bilirubin (TB).

**Conclusion:**

Even mild or asymptomatic SARS-CoV-2 infection is associated with subtle but consistent alterations in lipid metabolism and liver enzymes in ambulatory, unvaccinated individuals. These findings support the need for biochemical monitoring after SARS-CoV-2 infection, particularly in populations with high baseline cardiometabolic risk.

## Introduction

COVID-19 is a highly infectious disease caused by SARS-CoV-2 (severe acute respiratory syndrome coronavirus 2), characterized by a broad spectrum of clinical presentations ranging from asymptomatic infection to respiratory failure and death (1). Although the disease is not exclusive to any particular population, individuals with cardiovascular disease, type 2 diabetes, and obesity have been shown to be at higher risk of developing severe complications (2).

Since the beginning of the pandemic, numerous studies have highlighted the systemic effects of SARS-CoV-2 infection, which go beyond the respiratory tract and involve multiple metabolic and immunological pathways (3, 4). In hospitalized patients, significant alterations in biochemical and hematological markers have been reported, including changes in glucose, lipid profiles, liver enzymes, inflammatory markers, and complete blood counts (5–8). One of the most consistently altered biomarkers is high-density lipoprotein cholesterol (HDL-C), which has been associated with inflammation and worse outcomes (8, 9).

However, most of this evidence comes from inpatient cohorts, leaving an important gap in knowledge regarding these markers in ambulatory individuals or those with asymptomatic or mild disease. Moreover, most published studies have focused on populations from Europe, Asia, or North America, with limited data available from Latin America, particularly from central-northern Mexico. Considering that metabolic and immune responses may vary by region due to genetic, dietary, and environmental factors (10), region-specific evidence is needed.

To address these gaps in the existing literature, we conducted a cross-sectional study during the pre-vaccination phase of the pandemic (August to December 2020) in an ambulatory population from central-northern Mexico. The objective was to compare biochemical and hematological parameters between outpatients with and without anti–SARS-CoV-2 IgG and IgM antibodies, in order to explore potential subclinical alterations associated with prior virus exposure in a non-hospitalized population during the pre-vaccination phase.

## Methods

### Study population

A cross-sectional study was conducted on 201 individuals residing in the state of San Luis Potosí, Mexico. Eligible participants were ambulatory individuals aged ≥18 years. Exclusion criteria included pregnancy and substance abuse. Elimination criteria included voluntary withdrawal.

Participants were voluntarily recruited at the Universidad Autónoma de San Luis Potosí, and all provided written informed consent. The study was approved by the Committee for Ethics in Health Research (CEID2020-06R1).

### Variables

Independent variables were categorized into hematological and biochemical parameters, whereas the dependent variable was the presence of anti–SARS-CoV-2 antibodies (IgG and/or IgM antibodies).

### Sample collection and laboratory procedures

Venous blood samples were collected from participants following a 12-h fasting period. Serum was obtained by centrifugation under standardized conditions for biochemical analysis, while whole blood was used for hematological evaluation.

### Detection of IgG and IgM antibodies against SARS-CoV-2

The presence of anti–SARS-CoV-2 IgM and IgG antibodies was assessed using lateral flow chromatographic immunoassays: the Panbio™ COVID-19 IgG/IgM Rapid Test Device (Abbott, Germany) and the Certum IgG/IgM Rapid Test™ (All Test Biotech Co., Ltd., Hangzhou, China), following the manufacturers’ instructions. These rapid tests allow for the qualitative and differential detection of IgG and IgM antibodies against SARS-CoV-2. The Panbio™ assay is directed against the nucleocapsid (N) antigen, whereas the Certum IgG/IgM Rapid Test™ detects antibodies targeting both the spike (S) and nucleocapsid (N) antigens. Participants were classified as seropositive only when reactivity was simultaneously detected by both assays, a criterion considered indicative of reliable evidence of natural SARS-CoV-2 infection, either in a late stage or, in some cases, potentially active.

### Hematological profile

The following parameters were determined from whole blood using a CELL-DYN 22 Emerald® hematology analyzer (Abbott Laboratories, Chicago, IL, USA): leukocytes, lymphocytes, MID (monocytes, basophils, and eosinophils), neutrophils, erythrocytes, hemoglobin, hematocrit (HCT), mean corpuscular volume (MCV), mean corpuscular hemoglobin (MCH), mean corpuscular hemoglobin concentration (MCHC), red cell distribution width (RDW), and platelet count.

### Biochemical profile

The biochemical profile included the measurement of fasting glucose, total cholesterol (TC), HDL-C, low-density lipoprotein cholesterol (LDL-C), triglycerides (TG), serum creatinine (SC), uric acid (UA), aspartate aminotransferase (AST), alanine aminotransferase (ALT), alkaline phosphatase (ALP), and total bilirubin (TB). All analyses were performed using the VITROS-250® automated analyzer (Ortho Clinical Diagnostics, Raritan, NJ, USA), employing standardized *in vitro* diagnostic methods and VITROS Chemistry Products slides, in accordance with the manufacturer’s specifications.

### Statistical analysis

A descriptive statistical analysis was performed to calculate frequencies and percentages for categorical variables, as well as means or medians with corresponding measures of dispersion for continuous variables. The McNemar test was used to compare concordance between the two antibody detection brands. The distribution of continuous variables was assessed using the Kolmogorov–Smirnov test. Depending on the distribution, comparisons between groups were made using the Student’s t-test for independent samples or the Mann–Whitney U test. Statistical significance was set at *p* < 0.05. All analyses were conducted using SPSS Statistics® version 21 (IBM Corp., Armonk, NY, USA).

## Results

### Seroprevalence of SARS-CoV-2 antibodies

A total of 201 participants, with a median age of 36 years (range: 18–81), were evaluated. Among them, 45 individuals (22.3%) showed simultaneous reactivity in two different rapid lateral flow immunochromatographic assays: Panbio™ targeting the nucleocapsid (N) antigen and Certum IgG/IgM Rapid Test™ targeting both N and spike (S) antigens. Of these seropositive individuals, 13 (6.46%) were positive for IgG only, 21 (10.4%) for IgM only, and 11 (5.47%) for both IgG and IgM antibodies.

When stratified by sex, females (*n* = 122) exhibited a cumulative seroprevalence of 18.7% (*n* = 23), whereas males (*n* = 79) showed a seroprevalence of 27.8% (*n* = 22). However, chi-square analysis revealed no statistically significant association between sex and seropositivity to SARS-CoV-2 antibodies (*p* = 0.166) (Table 1).

**Table 1:** Seroprevalence of anti-SARS-CoV-2 IgG and IgM antibodies (*n* = 201).

Sex	*n*	IgG	IgM	IgG + IgM	Cumulative seroprevalence	*p*^1^
		*n* (%)	*n* (%)	*n* (%)	*n* (%)	
Female	122	7 (5.73)	14 (11.4)	2 (1.63)	23 (18.7)	0.166
Male	79	6 (7.59)	7 (8.86)	9 (11.4)	22 (27.8)	
Total	201	13 (6.46)	21 (10.4)	11 (5.47)	45 (22.3)	

### Hematological parameters by sex

A comparative analysis of hematological parameters between female and male participants revealed statistically significant differences in several variables. Males exhibited significantly higher mean values of erythrocytes, hemoglobin, and HCT compared to females (all *p* < 0.001). Additionally, MCHC was slightly higher in males (*p* = 0.014), whereas platelet count was significantly lower in males than in females (*p* = 0.020).

No statistically significant differences were observed in leukocyte count, lymphocytes, MID, neutrophils, MCV, MCH, or RDW between sexes (*p* > 0.05 for all comparisons) (Table 2).

**Table 2:** Characteristics in hematological variables (*n* = 201).

Variable	*n*	Mean or Median	SD or (minimum - maximum)	*p*^1^
Leukocytes (K/μL)				
Female	122	6.70	3–12	0.292
Male	79	6.50	4–14	
Total	201	6.60	3.1–13.7	
Lymphocytes (K/μL)				
Female	122	2.50	1–4	0.833
Male	79	2.55	1–4	
Total	201	2.50	0.8–4.3	
MID (K/μL)				
Female	122	0.50	0.3–0.9	0.494
Male	79	0.50	0.3–1.0	
Total	201	0.50	0.3–1.0	
Neutrophils (K/μL)				
Female	122	3.6	1–9	0.342
Male	79	3.3	1–11	
Total	201	3.5	0.7–11	
Erythrocytes (K/μL)				
Female	122	4.57	± 0.43	<0.001*
Male	79	5.11	± 0.52	
Total	201	4.77	± 0.54	
Hemoglobin (g/dL)				
Female	122	14.1	9–17	<0.001*
Male	79	16.1	12–20	
Total	201	14.7	9–20	
HCT (%)				
Female	122	42.6	29–91	<0.001*
Male	79	48.9	38–62	
Total	201	44.3	29–91	
MCV (fL)				
Female	122	93.6	60–110	0.220
Male	79	94.2	85–105	
Total	201	93.9	60–110	
MCH (pg)				
Female	122	30.6	18–65	0.069
Male	79	31.1	28–39	
Total	201	30.8	18–65	
MCHC (g/dL)				
Female	122	32.6	21–40	0.014*
Male	79	32.9	30–40	
Total	201	32.7	21–40	
RDW (%)				
Female	122	11.2	6–18	0.308
Male	79	11.3	6–14	
Total	201	11.2	6–18	
Platelets (K/μL)				
Female	122	260	± 70.49	0.020*
Male	79	237	± 62.26	
Total	201	251	± 68.03	

MID, monocytes, basophils, and eosinophils; HCT, hematocrit; MCV, mean corpuscular volume (fL); MCH, mean corpuscular hemoglobin; MCHC, mean corpuscular hemoglobin concentration; RDW, red blood cell distribution width; SD, standard deviation.

^1^Student *T*-statistic for independent samples or U-Mann Whitney statistic for unrelated data.

^*^Significant difference by sex at *p* < 0.05.

### Biochemical parameters by sex

Statistically significant sex differences were also observed in the biochemical profile (Table 3). Male participants exhibited higher median fasting glucose (*p* = 0.038) and significantly lower HDL-C levels compared to females (*p* < 0.001). Conversely, triglyceride levels were significantly higher in males (*p* = 0.011).

**Table 3:** Characteristics in biochemical variables (*n* = 201).

Variable	*n*	Mean or Median	SD or (minimum - maximum)	*p^1^*
Glucose (mg/dL)				
Female	122	90	73–218	0.038*
Male	79	95	56–264	
Total	201	91	56–264	
TC (mg/dL)				
Female	122	184	± 38.1	0.952
Male	79	185	± 37.0	
Total	201	185	± 37.6	
HDL-C (mg/dL)				
Female	122	47.7	25–99	<0.001*
Male	79	40.0	13–82	
Total	201	43.6	13–99	
LDL-C (mg/dL)				
Female	122	105	± 32.7	0.278
Male	79	112	± 34.9	
Total	201	108	± 33.5	
TG (mg/dL)				
Female	122	126	43–575	0.011*
Male	79	152	47–575	
Total	201	137	43–575	
SC (mg/dL)				
Female	122	0.72	± 0.12	<0.001*
Male	79	0.98	± 0.15	
Total	201	0.82	± 0.18	
UA (mg/dL)				
Female	122	4.30	1.6–7.6	<0.001*
Male	79	6.20	2.6–9.1	
Total	201	5.10	1.6–9.1	
AST (U/L)				
Female	122	23	14–70	<0.001*
Male	79	29	15–125	
Total	201	25	14–125	
ALT (U/L)				
Female	122	18	8–94	<0.001*
Male	79	32.5	13–238	
Total	201	24	8–238	
ALP (U/L)				
Female	122	77	37–207	0.453
Male	79	79	44–190	
Total	201	77	37–207	
TB (mg/dL)				
Female	122	0.6	0.3–1.5	<0.001*
Male	79	0.7	0.3–3.6	
Total	201	0.6	0.3–3.6	

TC, total cholesterol; HDL-C, high-density lipoprotein cholesterol; LDL-C, low-density lipoprotein cholesterol; TG, triglycerides; SC, serum creatinine; UA, uric acid; AST, aspartate aminotransferase; ALT, alanine aminotransferase; ALP, alkaline phosphatase; TB, total bilirubin; SD, standard deviation.1 Student T-statistic for independent samples or U-Mann Whitney statistic for unrelated data.

*Significant difference by sex at *p* < 0.05.

Renal function markers showed a clear sex-related pattern: males presented higher SC and UA levels (both *p* < 0.001). Regarding the hepatic profile, males exhibited higher median levels of AST and ALT compared to females (both *p* < 0.001). TB was also slightly higher in males (*p* < 0.001), whereas no significant differences were detected for TC, LDL-C, or ALP.

Overall, these findings indicate a more adverse biochemical risk profile in male participants, especially regarding lipid metabolism and liver function.

### Biochemical and hematological parameters in relation to anti-SARS-CoV-2 antibody status

When biochemical parameters were compared between participants with and without anti–SARS-CoV-2 antibodies (Table 4), significant differences were observed in specific markers. HDL-C levels were significantly lower in seropositive individuals compared to seronegatives in the total population (median: 40.5 mg/dL vs. 45.3 mg/dL; *p* = 0.009). This difference reached statistical significance in females (*p* = 0.042), but not in males.

**Table 4:** Characteristics in biochemical variables in subjects with presence or absence anti-SARS-CoV-2 antibodies (*n* = 201).

Variable	*n*	Presence (*n* = 45)	Absence (*n* = 156)	*p*^1^
Glucose (mg/dL)				
Female	122	90 (77–152)	90 (73–218)	0.305
Male	79	90 (77–264)	96 (56–142)	0.566
Total	201	90 (77–264)	91 (56–218)	0.564
TC (mg/dL)				
Female	122	184 ± 27.2	184 ± 40.3	0.973
Male	79	182 ± 35.3	186 ± 38	0.650
Total	201	183 ± 31.1	185 ± 39.4	0.752
HDL-C (mg/dL)				
Female	122	43.4 (26–82)	48.5 (25–99)	0.042*
Male	79	38.7 (18–71)	40 (13–82)	0.320
Total	201	40.5 (18–82)	45.3 (13–99)	0.009*
LDL-C (mg/dL)				
Female	122	113 ± 26.8	105 ± 33.8	0.328
Male	79	108 ± 35.3	113 ± 34.7	0.587
Total	201	110 ± 30.9	108 ± 34.2	0.672
TG (mg/dL)				
Female	122	122 (65–528)	128 (43–575)	0.908
Male	79	139 (63–482)	155 (47–575)	0.961
Total	201	132 (63–528)	138 (43–575)	0.790
SC (mg/dL)				
Female	122	0.76 ± 0.14	0.72 ± 0.12	0.120
Male	79	0.92 ± 0.16	0.99 ± 0.14	0.079
Total	201	0.84 ± 0.17	0.82 ± 0.18	0.442
UA (mg/dL)				
Female	122	4.3 (2.7–7.2)	4.3 (1.6–7.6)	0.950
Male	79	5.5 (3–8.2)	6.4 (2.6–9.1)	0.016*
Total	201	4.9 (2.7–8.2)	5.1 (1.6–9.1)	0.561
AST (U/L)				
Female	122	27 (16–70)	22.5 (14–63)	0.167
Male	79	33 (21–125)	28 (15–92)	0.032*
Total	201	30 (16–125)	24 (14–92)	0.007*
ALT (U/L)				
Female	122	27 (10–70)	17 (8–94)	0.018*
Male	79	40 (19–238)	30 (13–181)	0.041*
Total	201	32.5 (10–238)	20 (8–181)	0.001*
ALP (U/L)				
Female	122	80 (53–125)	76 (37–207)	0.439
Male	79	81 (50–190)	79 (44–136)	0.352
Total	201	80.5 (50–190)	78 (37–207)	0.202
TB (mg/dL)				
Female	122	0.50 (0.3–1.5)	0.60 (0.3–1.4)	0.103
Male	79	0.80 (0.4–3.6)	0.70 (0.3–2.0)	0.417
Total	201	0.60 (0.3–3.6)	0.60 (0.3–2.0)	0.893

Median (minimum-maximum). Mean ± standard deviation. TC, total cholesterol; HDL-C, high-density lipoprotein cholesterol; LDL-C, low-density lipoprotein cholesterol; TG, triglycerides; SC, serum creatinine; UA, uric acid; AST, aspartate aminotransferase; ALT, alanine aminotransferase; ALP, alkaline phosphatase; TB, total bilirubin; SD, standard deviation.

^1^Student T-statistic or U-Mann Whitney statistic.

*Significant difference by absence or presence of antibodies at *p* < 0.05.

Liver enzymes were also significantly higher among seropositive participants. AST levels were elevated in antibody-positive individuals in the total population (median: 30 U/L vs. 24 U/L; *p* = 0.007), with statistical significance observed in males (*p* = 0.032). Similarly, ALT levels were significantly higher in seropositive subjects overall (median: 32.5 U/L vs. 20 U/L; *p* = 0.001), with significant differences in both females (*p* = 0.018) and males (*p* = 0.041).

In contrast, no significant differences were detected between seropositive and seronegative individuals for fasting glucose, TC, LDL-C, TG, ALP, or TB.

Regarding renal biomarkers, UA levels were significantly lower in seropositive males compared to seronegative males (median: 5.5 mg/dL vs. 6.4 mg/dL; *p* = 0.016). No significant differences in SC or UA were observed in females or in the total population.

No statistically significant differences were found in any hematological parameters between participants with and without anti–SARS-CoV-2 antibodies (Table 5).

**Table 5:** Characteristics of hematological variables in subjects with presence or absence of anti-SARS-CoV-2 antibodies (*n* = 201).

Variable	*n*	Presence (*n* = 45)	Absence (*n* = 156)	*p^1^*
Leukocytes (K/μL)				
Female	122	6.9 (4.3–11.8)	6.8 (3.1–11.5)	0.532
Male	79	6.0 (3.7–13.7)	6.6 (4.3–9.7)	0.362
Total	201	6.4 (3.7–13.7)	6.7 (3.1–11.5)	0.953
Lymphocytes (K/μL)				
Female	122	2.6 (1.1–4.3)	2.5 (0.8–4.3)	0.170
Male	79	2.1 (1.6–3.5)	2.7 (0.8–3.7)	0.109
Total	201	2.6 (1.1–4.3)	2.6 (0.8–4.30)	0.892
MID (K/μL)				
Female	122	0.5 (0.3–0.8)	0.5 (0.3–0.9)	0.951
Male	79	0.5 (0.3–1)	0.5 (0.3–0.8)	0.133
Total	201	0.5 (0.3–1.0)	0.5 (0.3–0.9)	0.545
Neutrophils (K/μL)				
Female	122	3.3 (2.2–8.6)	3.7 (0.7–8.8)	0.911
Male	79	2.8 (1.4–11)	3.4 (1.8–6.3)	0.404
Total	201	3.2 (1.4–11)	3.6 (0.7–8.8)	0.544
Erythrocytes (K/μL)				
Female	122	4.6 ± 0.44	4.5 ± 0.4	0.526
Male	79	4.9 ± 0.57	5.1 ± 0.5	0.262
Total	201	4.7 ± 0.52	4.7 ± 0.5	0.953
Hemoglobin (g/dL)				
Female	122	13.7 (8.5–16.6)	14.1 (9.5–16.5)	0.504
Male	79	16.1 (13–17.6)	16.1 (12.1–20.2)	0.380
Total	201	14.9 (8.5–17.6)	14.7 (9.5–20.2)	0.911
HCT (%)				
Female	122	41.8 (28.9–51.2)	43 (32.4–91)	0.583
Male	79	46.8 (37.8–55.1)	49 (37.7–61.8)	0.193
Total	201	44.4 (28.9–55.1)	44.4 (32.4–91)	0.788
MCV (fL)				
Female	122	92.4 (60.4–102)	93.6 (67.3–109)	0.257
Male	79	94.5 (85.1–102)	94.2 (85.1–104)	0.557
Total	201	94.2 (60.4–102)	93.8 (67.3–109)	0.654
MCH (pg)				
Female	122	30.4 (17.8–37.6)	30.6 (19.7–65.2)	0.323
Male	79	31.3 (27.5–39.3)	30.9 (27.5–39.2)	0.530
Total	201	30.7 (17.8–39.3)	30.8 (19.7–65.2)	0.764
MCHC (g/dL)				
Female	122	32.4 (21.4–36.8)	32.6 (29.3–40)	0.721
Male	79	33.5 (30.3–39.6)	32.9 (31.7–38.3)	0.667
Total	201	32.7 (21.4–39.6)	32.7 (29.3–40)	0.983
RDW (%)				
Female	122	11.2 (7.2–18.4)	11.2 (5.9–16.9)	0.356
Male	79	11.1 (5.9–12.6)	11.3 (7.2–13.5)	0.349
Total	201	11.2 (5.9–18.4)	11.3 (5.9–16.9)	0.909
Platelets (K/μL)				
Female	122	282 ± 70.8	257 ± 68	0.137
Male	79	244 ± 76.4	238 ± 57	0.694
Total	201	264 ± 75.1	250 ± 65.2	0.218

Median (minimum-maximum); Mean ± standard deviation. MID, monocytes, basophils, and eosinophils; HCT, hematocrit; MCV, mean corpuscular volume (fL); MCH, mean corpuscular hemoglobin; MCHC, mean corpuscular hemoglobin concentration; RDW, red blood cell distribution width; SD, standard deviation.

^1^Student T-statistic or U-Mann Whitney statistic.

^*^Significant difference by absence or presence of antibodies at *p* < 0.05.

Overall, the most consistent biochemical alterations associated with prior SARS-CoV-2 exposure in this ambulatory population were reduced HDL-C levels and elevated aminotransferases.

## Discussion

In this study conducted in a pre-vaccination context in central-northern Mexico, we evaluated the biochemical and hematological profiles of outpatients with serological evidence of previous exposure to SARS-CoV-2. While no significant differences were found in hematological parameters, we observed notable alterations in several biochemical markers, particularly in lipid metabolism and liver function.

One of the main strengths of this study is its timing: all participants were recruited before COVID-19 vaccines became available, allowing us to observe the natural biochemical and metabolic response to SARS-CoV-2 infection in an unvaccinated ambulatory population. This is particularly important given emerging evidence that unvaccinated individuals who recover from COVID-19 may still develop new-onset metabolic disorders, such as dyslipidemia, even in mild cases (11). Moreover, persistence of biochemical alterations, such as elevated liver enzymes or changes in lipid profiles, has been described in Long COVID, especially among unvaccinated individuals, reinforcing the clinical value of our pre-vaccination data (12, 26). Studying an unvaccinated cohort thus provides valuable insight into subclinical or long-term sequelae in low-risk populations.

Our study demonstrated that HDL-C levels were significantly reduced and ALT and AST levels elevated in seropositive individuals, suggesting that even mild or asymptomatic SARS-CoV-2 infection can exert subclinical effects on lipid and liver metabolism. The reduction in HDL-C aligns with previous reports indicating that SARS-CoV-2–induced inflammation alters lipid handling, possibly through cytokine-mediated suppression of apolipoprotein A-I synthesis and enhanced catabolism of HDL-C particles (13, 14). These mechanisms may reduce the anti-inflammatory and antioxidant functions of HDL-C, contributing to systemic inflammation and potentially increasing cardiovascular risk in the post-infection phase (15, 27).

Elevated ALT and AST in seropositive individuals is consistent with hepatic involvement observed in COVID-19, even in non-hospitalized cohorts. Hepatic alterations may result from direct viral tropism via ACE2 expression in hepatocytes and cholangiocytes, as well as indirect effects of systemic inflammation and metabolic stress (16, 28). While most hospitalized patients present marked elevations in aminotransferases, our findings highlight that even ambulatory subjects may experience subclinical hepatic stress, reinforcing the need for monitoring liver function post-infection.

Interestingly, we observed sex-related differences in renal biomarkers, with seropositive males showing lower SC and UA compared to seronegatives. Although these differences were modest, they may reflect alterations in renal handling of metabolites during or after infection. Previous studies have described both hyperuricemia and hypouricemia during acute COVID-19, potentially linked to oxidative stress and changes in renal tubular transport (17). The directionality of our results, showing lower levels in seropositive males, suggests heterogeneity in renal metabolic adaptation after mild infection that warrants further study.

Contrary to many inpatient reports, our study found no significant differences in hematological parameters between seropositive and seronegative participants. In severe COVID-19, lymphopenia, neutrophilia, and elevated RDW are well-established prognostic markers (18, 19). However, our findings suggest that in ambulatory or mild cases, hematopoietic function is largely preserved. This highlights the importance of stratifying biomarker interpretation by disease severity.

Overall, our results support the notion that even mild SARS-CoV-2 infection may induce subtle biochemical alterations, particularly in lipid and liver metabolism, which could predispose individuals to long-term metabolic complications. These findings are especially relevant for Latin American populations, where the prevalence of obesity, diabetes, and metabolic syndrome is high (20, 29). Preventive follow-up of lipid and hepatic markers in post-COVID outpatients may be a cost-effective strategy to mitigate downstream cardiometabolic risks.

Our findings of decreased HDL-C and elevated ALT and AST in seropositive individuals are consistent with reports from other ambulatory cohorts worldwide. In a Spanish cohort study including outpatients with mild COVID-19, Masana et al. (14) described that low HDL-C and high triglycerides were frequent alterations, even in the absence of severe disease, reinforcing the notion that lipid metabolism is highly sensitive to SARS-CoV-2–induced inflammation. Similarly, a recent U. S. population-based study by Sorokin et al. (15) highlighted persistent dyslipidemia, particularly reduced HDL-C, as a common feature in post-COVID subjects, with potential implications for cardiovascular risk.

Evidence from Latin America also supports our observations. A Brazilian study reported lower HDL-C levels in recovered COVID-19 outpatients compared to controls, (21). These data align with our results and emphasize that populations in Latin America—already burdened by high prevalence of obesity and metabolic syndrome—may face an amplified risk for post-infection cardiometabolic complications.

International studies have reported that mild elevations in ALT and AST are not limited to severe COVID-19 cases but can also occur in asymptomatic or oligosymptomatic individuals (16, 22). Collectively, these findings suggest that hepatic involvement is a frequent yet often subclinical feature of COVID-19, reinforcing the need for biochemical follow-up even in patients who did not require hospitalization.

The identification of biochemical alterations in ambulatory, non-hospitalized individuals with anti-SARS-CoV-2 antibodies carries important clinical implications. First, the consistent reduction in HDL-C observed in our study suggests that SARS-CoV-2 infection may contribute to a pro-atherogenic lipid profile, even in individuals with mild or asymptomatic disease. This is particularly relevant in Latin American countries, such as Mexico, where the prevalence of obesity, metabolic syndrome, and type 2 diabetes is among the highest worldwide (23). The additive effect of COVID-19–related dyslipidemia on pre-existing metabolic risk factors may increase the likelihood of future cardiovascular complications in these populations.

Second, the elevation of ALT and AST in seropositive participants highlights the potential for subclinical hepatic involvement, reinforcing the need for long-term monitoring of liver function. Although most alterations remain within reference ranges, even mild enzyme elevations could indicate underlying hepatocellular stress or inflammation, which might predispose certain individuals to chronic hepatic conditions (16). Given the high burden of non-alcoholic fatty liver disease (NAFLD) in Mexico and other Latin American countries, SARS-CoV-2 infection could act as an additional stressor that accelerates disease progression (24).

From a preventive perspective, our findings support the incorporation of simple biochemical monitoring (lipid profile and hepatic enzymes) into follow-up protocols for patients with confirmed prior exposure to SARS-CoV-2, regardless of disease severity. Such an approach would allow early identification of individuals at risk and the implementation of lifestyle or pharmacological interventions to mitigate long-term complications. In particular, strategies aimed at improving HDL-C levels, such as dietary modification, physical activity, and smoking cessation, may play a protective role in this context.

The study has limitations. Pre-existing comorbidities were not included in the analytical models; therefore, residual confounding and potential effect modification cannot be excluded. Additionally, the time elapsed since SARS-CoV-2 infection could not be precisely determined or incorporated as a covariate. Given the cross-sectional design and serological classification in an ambulatory, non-hospitalized population, variability in this interval may have introduced residual heterogeneity in the observed biochemical parameters.

Although participants were recruited during a defined epidemiological period prior to the introduction of vaccination, the potential influence of infection timing cannot be entirely ruled out. In the current context of widespread vaccination and hybrid immunity, replication in strictly unvaccinated populations is inherently limited; however, analyses based on historical pre-vaccination cohorts may help strengthen the interpretation of these associations within the specific temporal framework of the study.

One of the main strengths of this study was the classification of seropositive individuals exclusively as those showing simultaneous reactivity in two rapid immunochromatographic assays from different manufacturers (Panbio™ and Certum IgG/IgM Rapid Test™). This dual-testing strategy increases diagnostic reliability and allows a more accurate association between observed biochemical alterations and the immune response derived from natural SARS-CoV-2 infection.

Previous studies have evaluated rapid immunochromatographic assays in comparison with automated immunoassays, reporting variable but generally acceptable sensitivity and specificity, supporting their use as complementary diagnostic tools (25). Moreover, serological testing is particularly valuable for confirming infection after the first week of symptom onset, when the sensitivity of molecular viral RNA detection declines, while antibody detection increases substantially.

Rapid serological assays remain valuable due to their ease of use, low cost, and rapid turnaround time, especially in regions with limited access to automated platforms or low vaccination coverage. Assays targeting the nucleocapsid (N) antigen offer an additional advantage by enabling differentiation between natural infection and vaccine-induced immune responses, as current vaccines primarily target the spike (S) protein (30). This reinforces their relevance not only for clinical assessment but also for epidemiological surveillance.

An additional strength of this study lies in its focus on ambulatory, non-hospitalized individuals and the comprehensive evaluation of biochemical and hematological parameters, allowing the identification of subtle alterations that might remain undetected in asymptomatic or mildly symptomatic patients. Furthermore, the inclusion of an unvaccinated population provides valuable baseline information to distinguish the direct metabolic impact of SARS-CoV-2 infection from vaccine-related effects.

Although global vaccination coverage is currently high, certain subpopulations without prior vaccination and settings with limited vaccine access persist. In such contexts, our findings may still offer clinically relevant insight into the metabolic consequences of natural SARS-CoV-2 infection. Moreover, historical pre-vaccination cohorts are essential for understanding the unmodified biological response to the virus and for contextualizing findings in populations with heterogeneous or hybrid immunity. These considerations underscore the importance of longitudinal studies in diverse immunological settings to clarify the persistence of these alterations and their relationship with long COVID.

## Data Availability

The raw data supporting the conclusions of this article will be made available by the authors, without undue reservation.
